# Why Does 2,3,5,6-Tetrachlorophenol Generate the Strongest Intrinsic Chemiluminescence among All Nineteen Chlorophenolic Persistent Organic Pollutants during Environmentally-friendly Advanced Oxidation Process?

**DOI:** 10.1038/srep33159

**Published:** 2016-10-17

**Authors:** Hui-Ying Gao, Li Mao, Bo Shao, Chun-Hua Huang, Ben-Zhan Zhu

**Affiliations:** 1 State Key Laboratory of Environmental Chemistry and Ecotoxicology, Research Center for Eco-Environmental Sciences, Chinese Academy of Sciences, Beijing 100085, P. R. China; 2 University of Chinese Academy of Sciences, Beijing 100049, P. R. China; 3 Linus Pauling Institute, Oregon State University, Corvallis, OR 97331, USA

## Abstract

We found recently that intrinsic chemiluminescence (CL) could be produced by all 19 chlorophenolic persistent organic pollutants during environmentally-friendly advanced oxidation processes. Interestingly and unexpectedly, the strongest CL was produced not by the most-highly chlorinated pentachlorophenol (PCP), but rather by the less chlorinated 2,3,5,6-tetrachlorophenol (2,3,5,6-TeCP), one of the three tetrachlorophenol (TeCPs) isomers. However, it remains unclear what is the underlying molecular mechanism. Here we show that not only chlorinated quinoid intermediates, but more interestingly, semiquinone radicals were produced during the degradation of the three TeCPs and PCP by Fenton reagents, and the type and yield of which were found to be well correlated with CL generation. We propose that hydroxyl radical-dependent formation of more tetrachlorinated quinoids, quinone-dioxetanes and electronically excited carbonyl species might be responsible for the exceptionally strong CL production by 2,3,5,6-TeCP as compared to PCP and its two isomers. This is the first report showing the critical role of quinoid intermediates and semiquinone radicals in CL generation from polychlorinated phenols and Fenton system. These new findings may have broad chemical and environmental implications for future studies on remediation of other halogenated persistent aromatic pollutants by advanced oxidation processes.

Chlorophenols have found wide use in pesticides, herbicides, disinfectants, wood preservatives, personal care formulations, dyestuff intermediates and many other products, and they are also substantial by-products of wood pulp bleaching with chlorine^1^,^2^. Chlorophenols, especially the highly chlorinated ones, persist for decades in the environment because of their resistance to microbiological degradation, leading to the accumulation of these toxic substances in living organisms^3^. Their accumulation in the environment has caused considerable damage and threat to the ecosystem in aquatic bodies and human health^4^. Pentachlorophenol (PCP), which has been used as a wood preservative worldwide and also to kill snails to prevent snail fever in developing countries, is the most toxic representative of the chlorophenols^5^,^6^. PCP has been found in over one-fifth of the National Priorities List sites identified by the US Environmental Protection Agencies (EPA), and listed by US and European EPA as priority pollutants^1^,^7^,^8^. Mixtures of tetrachlorophenols (TeCPs) are generally used at sawmills as wood preservatives. 2,3,5,6-TeCP has been found in at least 355 of the 848 hazardous substances listed by the Agency for Toxic Substances and Disease Registry (ATSDR)^9^. Moreover, PCP and 2,3,4,6-TeCP have been classified as group 2B environmental carcinogens by the International Association for Research on Cancer (IARC)^4^,^8^,^10^. Therefore, it is necessary to develop highly efficient and reliable technologies for their removal.

The catalytic decomposition of hydrogen peroxide (H_2_O_2_) by ferrous ion catalyst (i.e., Fenton reagent)^11^ is one of the most popular advanced oxidation processes (AOPs) for the treatment of wastewaters containing chlorophenols^12^. The major advantage of Fenton process is that the reagent components are easy to handle and environmentally benign.

Chemiluminescence (CL) is a phenomenon in which molecules in a chemically generated excited state liberate energy with light emission. It has been shown that CL often accompanies decomposition of organic peroxides and generation of free radicals^13^,^14^,^15^,^16^,^17^. CL intensity-based analytical assays are inherently highly sensitive, rapid, and simple to operate, without the need for pretreatment of sample. Therefore, they are being increasingly used as a sensitive analytical method in various research fields^18^,^19^.

During our study of metal-independent hydroxyl/alkoxyl/ketoxy radical production by halogenated quinones and H_2_O_2_ (or organic hydroperoxides)^20^,^21^,^22^,^23^,^24^,^25^, and their potential biological effects^26^, we found that unprecedented hydroxyl radical-dependent two-step chemiluminescence could be generated by polyhalogenated quinones and H_2_O_2_^23^. Recently, we also observed an intrinsic CL generation by all 19 chlorophenols (the parent compounds of chlorinated quinones) and the classic ^•^OH-generating Fenton system, even in the absence of fluorescent agents^27^. One notable general trend is that the CL intensity increases with increasing number of chlorine atoms on the phenolic ring. Interestingly and unexpectedly, the strongest CL emission was found to be produced by 2,3,5,6-TeCP, which is even stronger than the most-highly chlorinated PCP, and much more stronger than two other TeCP isomers (2,3,4,5-, and 2,3,4,6-TeCP)^27^. However, it remains unclear what is the underlying molecular mechanism.

Therefore, in this study, we addressed the following questions: (i) Whether are chlorinated quinoid intermediates and/or semiquinone radicals produced during the degradation of chlorophenols by Fenton system; (ii) If so, are there any correlations between the yields and types of these quinoid intermediates and/or semiquinone radicals and CL production; (iii) What is the underlying molecular basis for this correlation?

## Results and Discussion

### The exceptionally strong intrinsic CL emission was produced by 2,3,5,6-tetrachlorophenol (2,3,5,6-TeCP), which is even stronger than the most-highly chlorinated PCP, and much more stronger than two other TeCP isomers

**Figure 1:**
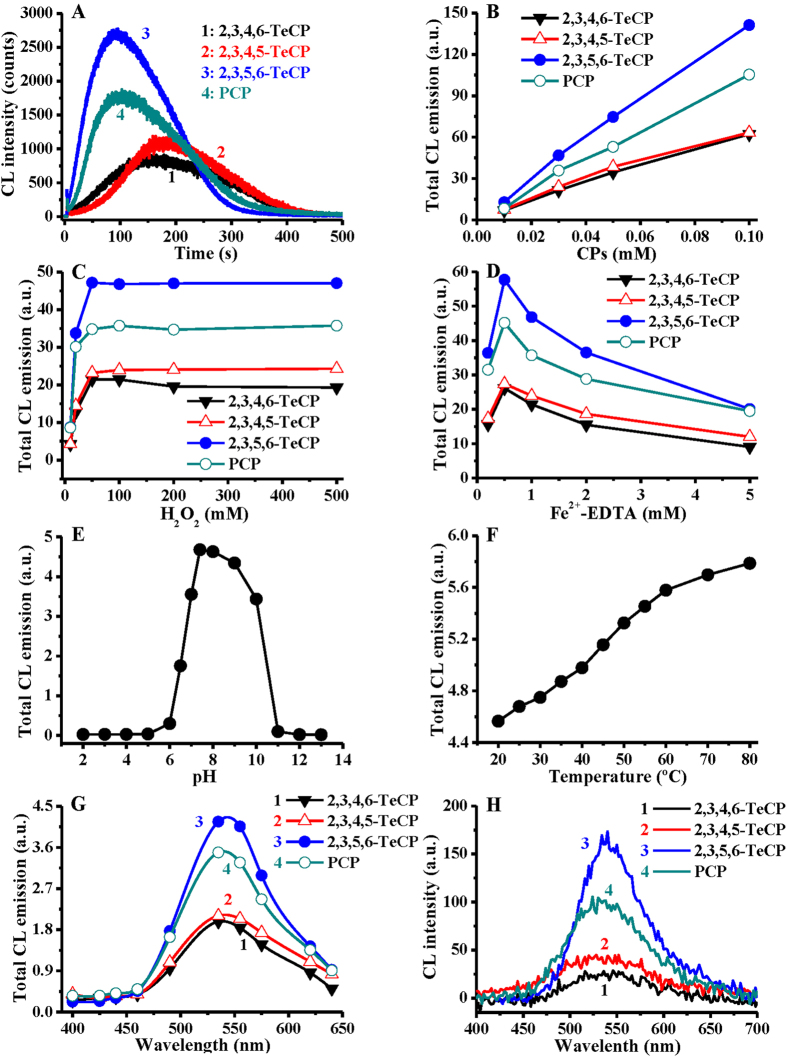
The CL emission could be produced during the degradation of TeCPs and PCP by Fenton system. (**A**) CL could be produced by TeCPs and PCP/Fenton system. TeCPs and PCP, 0.03 mM; H_2_O_2_, 100 mM; Fe^2+^-EDTA, 1 mM. (**B–D**) The dose-dependent effects of chlorophenols, H_2_O_2_ and Fe^2+^-EDTA on CL production by chlorophenols/Fenton. TeCPs and PCP, 0.01–0.1 mM; H_2_O_2_, 10–500 mM; Fe^2+^-EDTA, 0.2–5 mM. (**E,F**) The effect of pH and temperature on CL production by 2,3,5,6-TeCP/Fenton. 2,3,5,6-TeCP, 0.03 mM; H_2_O_2_, 100 mM; Fe^2+^-EDTA, 1 mM. (**G**) The CL emission spectra measured by static-injection ultraweak CL analysis. TeCPs and PCP, 0.03 mM; H_2_O_2_, 100 mM; Fe^2+^-EDTA, 1 mM. (**H**) The CL emission spectra measured by spectrofluorometry. TeCPs and PCP, 2 mM; H_2_O_2_, 1000 mM; Fe^2+^-EDTA, 5 mM. All reactions were carried out in chelex-pretreated phosphate buffer (0.1 M, pH 7.4).

As mentioned above, we found recently that intrinsic CL could be produced by all 19 chlorophenol congeners and Fenton reagent, which was further confirmed under a different experimental condition in this study (Supplementary Fig. S1A). Consistent with the results in our earlier report^27^, the strongest CL was produced not by the most-highly chlorinated PCP, but rather by the less chlorinated 2,3,5,6-TeCP, one of the three tetrachlorophenol isomers (Fig. 1A).

To guarantee the above findings were not just a special phenomenon under a specific experimental condition, various different experimental conditions were further investigated, especially for the three groups of highly chlorinated phenols (trichlorophenols (TCPs), TeCPs, and PCP), and analogous results were also observed (Supplementary Fig. S1B–D), which re-confirmed our previous findings. Therefore, it is interesting to know why such an exceptional CL phenomenon exists, and what is the underlying molecular mechanism?

To further investigate this unusual CL phenomenon and its underlying molecular mechanism, 2,3,5,6-TeCP was chosen as a model chlorophenol for more detailed studies. In comparison, PCP and the two other TeCP isomers (2,3,4,5-, and 2,3,4,6-TeCP) were studied in parallel.

We found that the intensity of CL emission was dependent on the concentrations of both chlorophenols and Fenton reagent (Fig. 1B–D). The CL produced by 2,3,5,6-TeCP/Fenton system was also found to depend on pH of the buffer: No CL was observed at pH ≤ 5; as the pH increased, the intensity of CL increased progressively, and reached maximum at pH 7.4-8; further increase of pH, however, led to a decline of CL intensity (Fig. 1E). A good correlation was observed between CL generation, 2,3,5,6-TeCP degradation and ^•^OH production at various pH (Supplementary Fig. S2). The higher the yield of ^•^OH, the faster the rate of 2,3,5,6-TeCP degradation, and the stronger the CL emission. We also found that the CL intensity was temperature-dependent: the higher the temperature in the range of 15 °C to 80 °C, the stronger the CL intensity (Fig. 1F). The CL emission spectra of TeCPs and PCP/Fenton system were obtained by both ultraweak CL analyzer and fluorospectrometer, respectively (Fig. 1G,H). Analogous CL emission spectra were observed for these four chlorinated phenols, with the maximum CL emission at a broad band 535–555 nm.

It should be noted that the CL produced by 2,3,5,6-TeCP/Fenton system was also directly dependent on ^•^OH formation, as indicated by the following lines of evidence: (i) The CL produced by 2,3,5,6-TeCP/Fenton system was markedly inhibited by several typical ^•^OH scavengers, such as dimethyl sulfoxide (DMSO), benzoate, and L-cysteine (Supplementary Fig. S3A), and the concentrations for each ^•^OH scavenger to inhibit 50% of CL were found to be 0.58, 0.33, and 0.15 mM, respectively; (ii) not only the CL reaction and ^•^OH-generation could be markedly accelerated but also the CL intensity and ^•^OH yield were enhanced significantly as the concentration of H_2_O_2_ was increased (Fig. 1C and Supplementary Fig. S8E); (iii) CL was also observed when Fe^2+^-EDTA was substituted by two other well-known ^•^OH-generating Fenton agents Fe^2+^-DTPA (diethylenetriaminepentaacetic acid) and Fe^2+^-NTA (nitrilotriacetic acid) (Supplementary Fig. S3B);^11^,^28^ and (iv) CL could be produced not only with the classic Fe^2+^-mediated Fenton systems, but also with other redox-active metal-mediated Fenton-like systems including cobalt(II)-EDTA/H_2_O_2_, chromium(III)-EDTA/H_2_O_2_ and vanadyl(II)-EDTA/H_2_O_2_, which could all produce ^•^OH (Supplementary Fig. S3C). Interestingly, the CL trend of TeCPs and PCP in all these ^•^OH-generating systems was exactly identical to the classic Fenton system.

### Chlorinated quinoid compounds were found as the major reaction intermediates during the degradation of TeCPs and PCP by Fenton reagent

To further investigate the underlying mechanism of the above interesting findings, the time course of CL emission and degradation of 2,3,5,6-TeCP by Fenton system was investigated, and we found that the CL emission could not reach maximum until 2,3,5,6-TeCP was almost completely degraded (more than 95%) (Supplementary Fig. S4). Interestingly, the CL emission spectrum of 2,3,5,6-TeCP/Fenton system was also found to be well correlated with the CL spectrum of tetrachloro-*p*-benzoquinone (*P*-TCBQ) and H_2_O_2_ as we previously reported^23^. These results suggested that the species responsible for CL emission should not be 2,3,5,6-TeCP itself, but probably its quinoid degradation intermediates.

**Figure 2:**
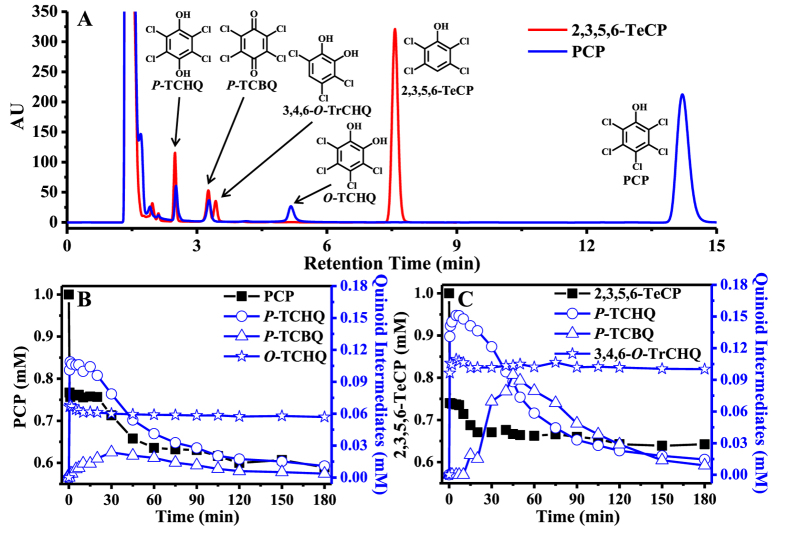
Identification (**A**) and quantification (**B,C**) of the major chlorinated quinoid intermediateds from the reaction of 2,3,5,6-TeCP and PCP with the Fenton system by HPLC analysis. For the separation and identification of chlorophenols and chlorinated quinoid intermediates: PCP/2,3,5,6-TeCP, 1 mM; H_2_O_2_, 1 mM; Fe^2+^-EDTA, 3 mM. All reactions were carried out in chelex-pretreated phosphate buffer (0.1 M, pH 7.4) at 25 °C. PCP, pentachlorophenol; 2,3,5,6-TeCP, 2,3,5,6-tetrachlorophenol; *P*-TCHQ, tetrachloro-*p*-hydroquinone; *P*-TCBQ, tetrachloro-*p*-benzoquinone; *O*-TCHQ, tetrachloro-*o*-hydroquinone; 3,4,6-*O*-TrCHQ, 3,4,6-trichloro-*o*-hydroquinone.

It has been shown that chlorophenols can be oxidized by ^•^OH during advanced oxidation processes (AOPs) to produce several chlorinated quinoid intermediates, which include chloro-*p*-hydroquinones (*P*-CHQs), chloro-*p*-benzoquinones (*P*-CBQs), chloro-*o*-hydroquinones (*O*-CHQs) and chloro-*o*-benzoquinones (*O*-CBQs)^27^,^29^,^30^. Indeed, we found that this is the case in our study. The major reaction intermediates produced by TeCPs and PCP were identified by HPLC-UV method with the respective authentic compounds as standards. As expected, we found that three of these four chlorinated quinoid compounds, including *P*-CHQs, *P*-CBQs and *O*-CHQs, are indeed the major quinoid degradation intermediates of the three TeCPs and PCP (Fig. 2 and Supplementary Fig. S5). For example, PCP could produce *P*-TCBQ, tetrachloro-*p*-hydroquinone (*P*-TCHQ), and tetrachloro-*o*-hydroquinone (*O*-TCHQ); 2,3,4,6-TeCP could produce trichloro-*p*-benzoquinone (*P*-TrCBQ), trichloro-*p*-hydroquinone (*P*-TrCHQ), 3,4,6-trichloro-*o*-hydroquinone (3,4,6-*O*-TrCHQ), and 3,4,5-trichloro-*o*-hydroquinone (3,4,5-*O*-TrCHQ). However, *O*-CBQs were not detected, possibly due to their extreme instability. Based on the formation of the same chlorinated quinoid compounds *P*-CHQs/*P*-CBQs, the four chlorinated phenols can be divided into two different groups. One group includes PCP and 2,3,5,6-TeCP, which could produce *P*-TCHQ/*P*-TCBQ; the other group includes 2,3,4,5- and 2,3,4,6-TeCP, which could produce *P*-TrCHQ/*P*-TrCBQ. Interestingly, the formation kinetics of these major quinoid intermediates correlated well with the degradation of TeCPs and PCP (Fig. 2 and Supplementary Fig. S5).

### A good correlation was observed between CL emission and the formation of the chlorinated quinoid intermediates

**Figure 3:**
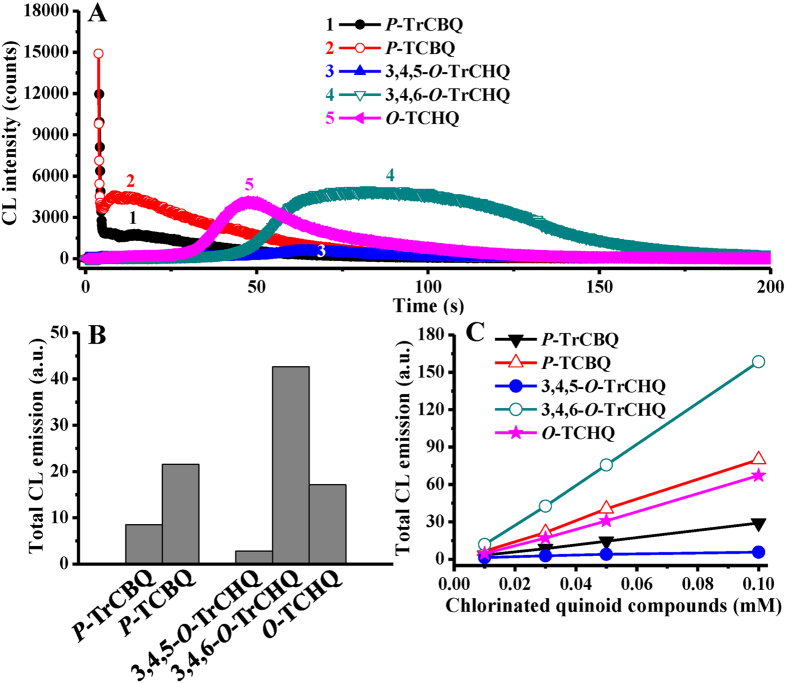
CL emission from the major chlorinated quinoid compounds *P*-CBQs and *O*-CHQs in Fenton system. (**A**) The CL emission of *P*-CBQs and *O*-CHQs in Fenton system. *P*-CBQs and *O*-CHQs, 0.03 mM; H_2_O_2_, 100 mM; Fe^2+^-EDTA, 1 mM. (**B**) The CL trend of *P*-CBQs and *O*-CHQs. *P*-CBQs and *O*-CHQs, 0.03 mM; H_2_O_2_, 100 mM; Fe^2+^-EDTA, 1 mM. (**C**) The dose-dependent effect of *P*-CBQs and *O*-CHQs on CL emission. *P*-CBQs and *O*-CHQs, 0.01–0.1 mM; H_2_O_2_, 100 mM; Fe^2+^-EDTA, 1 mM. All reactions were carried out in chelex-pretreated phosphate buffer (0.1 M, pH 7.4) at 25 °C.

As mention above, we found recently that CL could be produced by *P*-CBQs in the presence of H_2_O_2_, which can be further enhanced remarkably by addition of Fe^2+^-EDTA^23^. In contrast, *O*-CHQs could not produce CL in the presence of only H_2_O_2_; however, they can also produce CL when both H_2_O_2_ and Fe^2+^-EDTA are present together. In the present study, we found that, even under various different experimental conditions, (i) the CL emission of *P*-CBQs increased with increasing number of chlorine atoms on the quinone ring (i.e., *P*-TCBQ > *P*-TrCBQ); (ii) the CL emission were especially strong for *O*-TCHQ and 3,4,6-*O*-TrCHQ, but much weaker for 3,4,5-*O*-TrCHQ (i.e., the order of CL emission for the chlorinated quinoid intermediates: 3,4,6-*O*-TrCHQ > *O*-TCHQ ≫3,4,5-*O*-TrCHQ) (Fig. 3). Therefore, we speculated that the CL intensity of TeCPs and PCP might be mainly dependent on the yields and types of the *P*-CBQs (since *P*-CHQs can also be further oxidized to form *P*-CBQs) with higher degree of chlorination (*P*-TCBQ vs *P*-TrCBQ), and that of the *O*-CHQs (3,4,6-*O*-TrCHQ/*O*-TCHQ vs 3,4,5-*O*-TrCHQ). As discussed below, this was found to be indeed the case.

**Figure 4:**
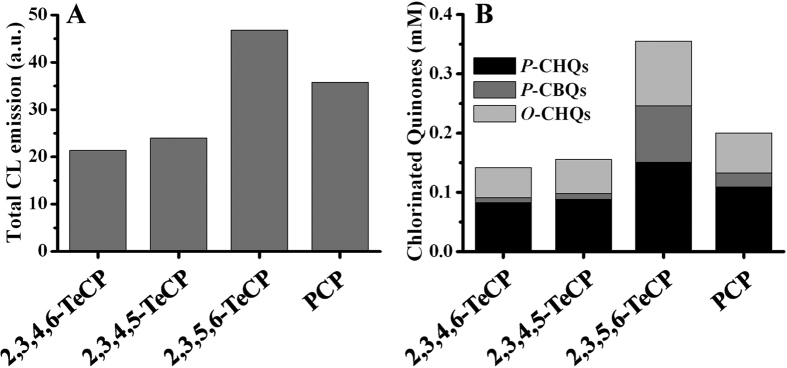
A Good correlation was observed between the total yields of *P*-CHQs/*P*-CBQs and *O*-CHQs, and the CL emission produced by TeCPs and PCP in Fenton system. (**A**) CL emission from TeCPs and PCP. TeCPs and PCP, 0.03 mM; H_2_O_2_, 100 mM; Fe^2+^-EDTA, 1 mM. (**B**) The total yields of *P*-CHQs/*P*-CBQs and *O*-CHQs. TeCPs and PCP, 1 mM; H_2_O_2_, 1 mM; Fe^2+^-EDTA, 3 mM. The reactions were carried out in chelex-pretreated phosphate buffer (0.1 M, pH 7.4) at 25 °C.

The maximum yields of each quinoid intermediate produced by TeCPs and PCP were summarized in Supplementary Table S1. We found that there is a good correlation between CL production and the total yields of their corresponding quinoid intermediates *P*-CHQs/*P*-CBQs and *O*-TCHQ/3,4,6-*O*-TrCHQ (the two *O*-CHQs with the stronger CL emission) produced by the two groups of chlorophenols (Fig. 4).

Then the question is why such a correlation exists?

From the above studies, two general principles were observed on CL production from *P*-CBQs and *O*-CHQs in Fenton system: (I) Among the *P*-CBQs and *O*-CHQs, *P*-TCBQ, *O*-TCHQ and 3,4,6-*O*-TrCHQ produced relative stronger CL emission; (II) For the same *P*-CBQs and *O*-CHQs, the higher yields of *P*-CBQs and *O*-CHQs were produced, the stronger the CL emission. Interestingly, the trend of CL emission from the three TeCPs and PCP can be well explained by the above two basic principles (Supplementary Table S2). For example, the CL intensity of the three TeCPs and PCP increases in the following order: 2,3,4,6-TeCP < 2,3,4,5-TeCP < PCP < 2,3,5,6-TeCP. This is because the yield of *P*-TCBQ/*P*-TCHQ produced by 2,3,5,6-TeCP is higher than that by PCP; in addition, the CL emission of 3,4,6-*O*-TrCHQ produced by 2,3,5,6-TeCP was stronger than that of *O*-TCHQ produced by PCP. Although 3,4,6-*O*-TrCHQ could also be produced by 2,3,4,6-TeCP, the yields of *P*-TrCBQ/*P*-TrCHQ and *O*-TCHQ produced by 2,3,4,5-TeCP were all higher than those of *P*-TrCBQ/*P*-TrCHQ and 3,4,6-*O*-TrCHQ produced by 2,3,4,6-TeCP, respectively. Therefore, when taking into account of both the yields and the types of *P*-CHQs/*P*-CBQs and *O*-CHQs produced by TeCPs and PCP with Fenton system, the unusual CL emission by 2,3,5,6-TeCP can be readily explained.

### Possible molecular basis for the correlation between CL and chlorinated quinoid intermediates

As mentioned above, we found that the CL generation from TeCPs and PCP/Fenton system correlated well with the formation of different types of quinoid intermediates and their yields. Now the question is:

What is the underlying molecular basis for such a correlation? When ^•^OH attacks on chlorophenols, the types and yields of the chlorinated quinoid intermediates (*P*-CHQs/*P*-CBQs and *O*-CHQs) were determined by the basic physiochemical properties of chlorophenols, which include the electron-withdrawing properties of Cl atoms and the directing effects of -OH and -Cl groups as listed below: (i) As the number of electron-withdrawing chloro substituent increases, the π-electron density in aromatic ring decreases, and as a result, it makes the phenolic ring less favorable for electrophilic (^•^OH) attack; however, it makes the quinone ring more favorable for nucleophilic attack by H_2_O_2_;^21^ (ii) Although both -OH and -Cl are ortho- and para- directing groups for further substitution or addition by ^•^OH, -OH is much stronger than -Cl; If the directing effects of -OH and -Cl groups are opposite to each other, the more powerful activating -OH group should play the dominant role;^31^ (iii) When the chlorine substituents were at the meta positions of the phenolic ring, the directing effects of -OH and meta-Cl will reinforce each other, and the reactions with ^•^OH should be highly regioselective for ortho- and para-positions; and (iv) ^•^OH attacks more readily on positions not occupied by -Cl groups. It is well known that the more negative charge density of C atom on the aromatic ring, the more favorable the electrophilic ^•^OH attacks it. Density functional theory (DFT) calculations were conducted on the atomic polar tensors (APT) charge for TeCPs and PCP with the B3LYP/6-311 + G* method. As can be seen from Supplementary Table S3, the charge density of the free positions are indeed more negative than that of positions occupied by -Cl. It has also been found^32^ that the dechlorination of polychlorinated dibenzo-*p*-dioxins attacted by ^•^OH would need higher activation energy than the reactions without dechlorination.

When the electrophilic ^•^OH attacks on chlorophenols, it is expected to attack the electron-rich positions, leading to the generation of mainly para- or ortho-OH-substituted quinoid intermediates (i.e., *P*-CHQs and *O*-CHQs, respectively). This was found to be the case in this study. Only three types of chlorinated quinoid intermediates can be detected due to the strong directing effect of -OH group (Supplementary Fig. S6). The formation ratios of *P*-CHQs/*O*-CHQs produced by TeCPs and PCP are all greater than 1:1. These results demonstrate that it is more favorable for ^•^OH substitution or addition at the para-position than at ortho-position under our experimental conditions. Interestingly, we also found that ^•^OH attack more easily at the ortho- and para-positions which are not occupied by chlroine atoms, which is consistent with previous literature reports^33^,^34^,^35^,^36^,^37^. For example, the yield of *P*-TCBQ/*P*-TCHQ produced by 2,3,5,6-TeCP with free position-4 is higher than that by PCP with full chloro-substitution. This is also one of the major reasons why the CL of the less chlorinated 2,3,5,6-TeCP is stronger than that of PCP. For 2,3,4,5-TeCP, the yield of *O*-TCHQ produced by ^•^OH attack at free 6-position is higher than that of 3,4,5-*O*-TrCHQ produced by ^•^OH attack at occupied 2-position.

### Chlorinated *p*-semiquinone radicals were unequivocally identified, for the first time, as major radical intermediates under the same experimental conditions for CL generation, by complementary application of both UV-visible spectrometric methods coupled with stopped-flow technique and direct ESR

As discussed above, *P*-TCHQ and *P*-TCBQ were found to be the major quinoid intermediates during the degradation of both 2,3,5,6-TeCP and PCP by Fenton reagent, which are consistent with previous reports of PCP oxidation by other advanced oxidation processes (ozonation and photocatalytic oxidation)^38^,^39^,^40^. It is well-known that the cyclic (auto)oxidation and reduction reactions of *P*-TCHQ and *P*-TCBQ can produce the intermediary of tetrachloro-*p*-semiquinone radical (*P*-TCSQ^•−^)^1^,^41^. However, to our knowledge, there was no evidence of *P*-TCSQ^•−^ formation during the degradation of PCP by Fenton reagent^42^,^43^, although *P*-TCSQ^•−^ can be detected in other ^•^OH-generating systems^39^,^40^,^44^. Therefore, it is interesting to know whether *P*-TCSQ^•−^ can be indeed produced in chlorophenols/Fenton system; and if so, is there any correlation between *P*-TCSQ^•−^ formation and CL generation?

In the present study, only under low concentrations of Fenton reagent could we successfully detect tetrachloro-*p*-semiquinone anion radical (*P*-TCSQ^•−^) formation, which shows a typical UV-visible spectrum with a maximum absorption peak at 453 nm and an ESR signal with *g* value at 2.0054^41^ (Supplementary Fig. S7 and Supplementary Table S4). We also found that there is a good correlation between *P*-TCSQ^•−^ formation and ^•^OH production by Fenton system (as measured by fluorescent method with terephthalic acid (TPA) as an ^•^OH probe): the more ^•^OH produced, the faster the formation of *P*-TCSQ^•−^ (Supplementary Fig. S8). Furthermore, the maximum yield of *P*-TCSQ^•−^ produced was also found to depend on pH of the buffer; and a good correlation was observed between the yield of *P*-TCSQ^•−^ and CL emission with varying pH (from 2 to 13) (Supplementary Fig. S9). These results suggest that the CL production by 2,3,5,6-TeCP and PCP/Fenton system might be directly dependent on the formation of their corresponding anion radical intermediate *P*-TCSQ^•−^.

**Figure 5:**
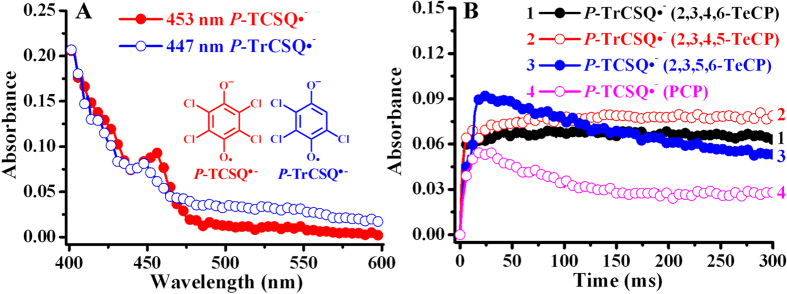
Transient *P*-CSQs^•−^ production by TeCPs and PCP/Fenton system under the same experimental conditions for CL generation. (**A**) Transient absorption spectra of *P*-CSQs^•−^. (**B**) The kinetics of *P*-TCSQ^•−^ formation at 453 nm and *P*-TrCSQ^•−^ at 447 nm. TeCPs and PCP, 0.5 mM; H_2_O_2_, 100 mM; Fe^2+^-EDTA, 1 mM. All reactions were carried out in chelex-pretreated phosphate buffer (0.1 M, pH 7.4) at 25 °C.

Interestingly, not only PCP and 2,3,5,6-TeCP could produce *P*-TCSQ^•−^, the other two TeCPs could also produce their corresponding chloro-*p*-semiquinone radicals (*P*-CSQs^•−^) in ^•^OH-generating Fenton system. Indeed, the same trichloro-*p*-semiquinone radical (*P*-TrCSQ^•−^) with the distinctive absorption spectrum was observed from 2,3,4,5- and 2,3,4,6-TeCP/Fenton system by UV-visible method (Supplementary Fig. S7). It should be noted that the rate of *P*-CSQs^•−^ formation increased with increasing concentration of chlorophenols and H_2_O_2_, but with decreasing concentration of Fe^2+^-EDTA (Supplementary Fig. S10). However, when we did the above experiments under the same experimental conditions as that for CL production (i.e., under the high concentrations of Fenton reagent), *P*-CSQs^•−^ cannot be detected by the conventional UV-visible and ESR methods. This is possibly due to the rapid formation and decay of *P*-CSQs^•−^ under these conditions. To solve this problem, stopped-flow technique, which is suitable for the kinetic study of fast reactions, was employed. Using this method, we can clearly observe the typical UV-visible spectra of *P*-TCSQ^•−^ and *P*-TrCSQ^•−^ generation from TeCPs and PCP/Fenton system under the real CL-producing experimental conditions (Fig. 5A). As expected, the two *P*-CSQs^•−^ were fleetingly formed and rapidly decayed (Fig. 5B). For example, the time of the maximum yield/disappearance of *P*-TCSQ^•−^ produced by PCP and 2,3,5,6-TeCP was 14 ms/120 ms and 26 ms/584 ms, respectively; and that of *P*-TrCSQ^•−^ produced by 2,3,4,5- and 2,3,4,6-TeCP was 140 ms/650 ms and 40 ms/402 ms, respectively.

**Figure 6:**
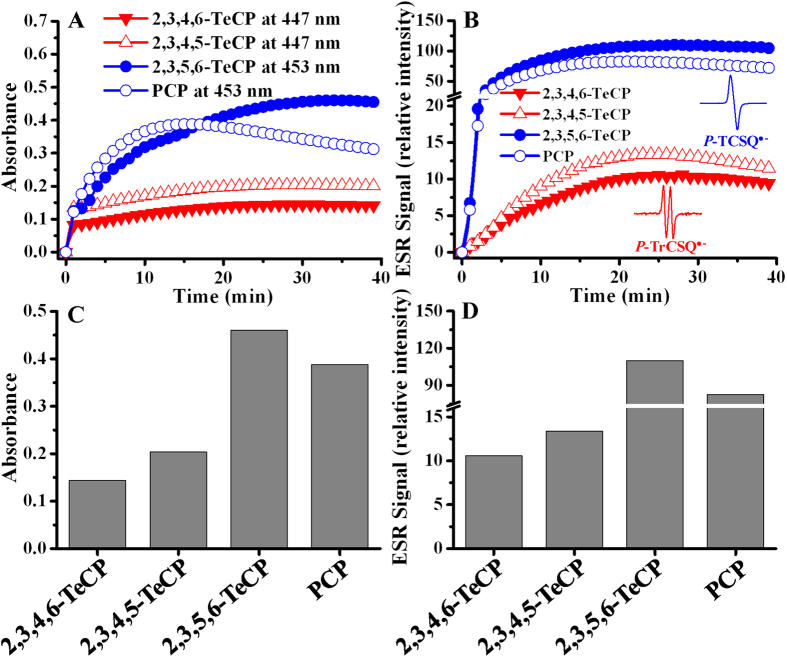
The kinetics of *P*-TCSQ^•−^ and *P*-TrCSQ^•−^ formation and decay in TeCPs and PCP/Fenton system by both UV-visible (**A,C**) and direct ESR (**B,D**) spectrometric methods. TeCPs and PCP, 0.5 mM; H_2_O_2_, 0.5 mM; Fe^2+^-EDTA, 1.5 mM. All reactions were carried out in chelex-pretreated phosphate buffer (0.1 M, pH 7.4) at 25 °C.

Then, the formation of *P*-CSQs^•−^ was further confirmed by direct ESR method, and the typical ESR spectra of *P*-CSQs^•−^ were obtained as well (Supplementary Table S4). Based on the formation of the same *P*-CSQs^•−^ intermediates, TeCPs and PCP can also be classified into two sub-groups (*P*-TrCSQ^•−^ and *P*-TCSQ^•−^), which agree well with the above proposed two sub-groups based on the formation of the same quinoid intermediates (*P*-CHQs/*P*-CBQs). For example, both *P*-TCSQ^•−^ (*g* = 2.0054; *λ*_max_ = 453 nm) and *P*-TCHQ/*P*-TCBQ can be produced by PCP and 2,3,5,6-TeCP; and both *P*-TrCSQ^•−^ (*a*^H^ = 2.17 G, *g* = 2.0051; *λ*_max_ = 447 nm) and *P*-TrCHQ/*P*-TrCBQ can be produced by 2,3,4,5-TeCP and 2,3,4,6-TeCP. Subsequently, the kinetics of *P*-TCSQ^•−^ and *P*-TrCSQ^•−^ formation and decay in TeCPs and PCP/Fenton system were investigated by both UV-visible and direct ESR spectrometric methods (Fig. 6A,B), and the maximum yields of each *P*-CSQs^•−^ were summarized (Fig. 6C,D). Due to the difference in the ESR responses of the various *P*-CSQs^•−^, the signal intensity of *P*-TCSQ^•−^ produced by 2,3,5,6-TeCP and PCP was far stronger than that of *P*-TrCSQ^•−^ produced by 2,3,4,6- and 2,3,4,5-TeCP under the same experimental conditions. Interestingly, very good correlations were observed between the maximum yields of *P*-CSQs^•−^ and the general trend of CL produced by the corresponding chlorophenols.

It should be noted that only one kind of semiquinone radical intermediates i.e., *P*-CSQs^•−^, was detected, but no ESR signals of chloro-*o*-semiquinone radicals (*O*-CSQs^•−^) were observed in this study. This could be attributed to the weak ESR signal and the instability of *O*-CSQs^•−^. For example, the signal intensity of *P*-TCSQ^•−^ (*g* = 2.0054) produced by *P*-TCHQ was found to be more than 150 times stronger than that of tetrachloro-*o*-semiquinone radicals (*O*-TCSQ^•−^, *g* = 2.0054) produced by *O*-TCHQ under the same experimental conditions (Supplementary Fig. S11). When the concentrations of *O*-TCHQ and *P*-TCHQ are in the same order of magnitude, the ESR signal of *O*-TCSQ^•−^ would be completely overlapped and overshadowed by that of *P*-TCSQ^•−^. Therefore, only *P*-TCSQ^•−^ could be observed.

In summary, we found that there is a good correlation between CL production and the formation of the chlorinated semiquinone radicals and/or the quinoid intermediates for TeCPs and PCP/Fenton system.

### Molecular mechanism of ^•^OH-dependent CL production by the highly chlorinated phenols and Fenton reagent

Besides the three chloroquinoid intermediates (*P*-CBQs, *P*-CHQs and *O*-CHQs) and semiquinone radicals, several hydroxylated chloroquinones and ring-opened compounds were also identified as degradation intermediates or final products of TeCPs and PCP by HPLC, ion chromatography (IC) and total organic carbon (TOC) analysis. For example, trichlorohydroxy-1,4-benzoquinone (TrCBQ-OH) and 2,5-dichloro-3,6-dihydroxy-1,4-benzoquinone (DDBQ), the further hydroxylation products of *P*-TCBQ/*P*-TCHQ/*O*-TCHQ, were identified in 2,3,5,6-TeCP/Fenton system. These hydroxylated chloroquinone intermediates could be further decomposed to the ring-opened products, such as dichloromaleic acid (DCMA), chloromalonic acid (CMA), oxalic acid (OA), formic acid (FA) and CO_2_ (and/or CO) (Supplementary Fig. S12).

**Figure 7:**
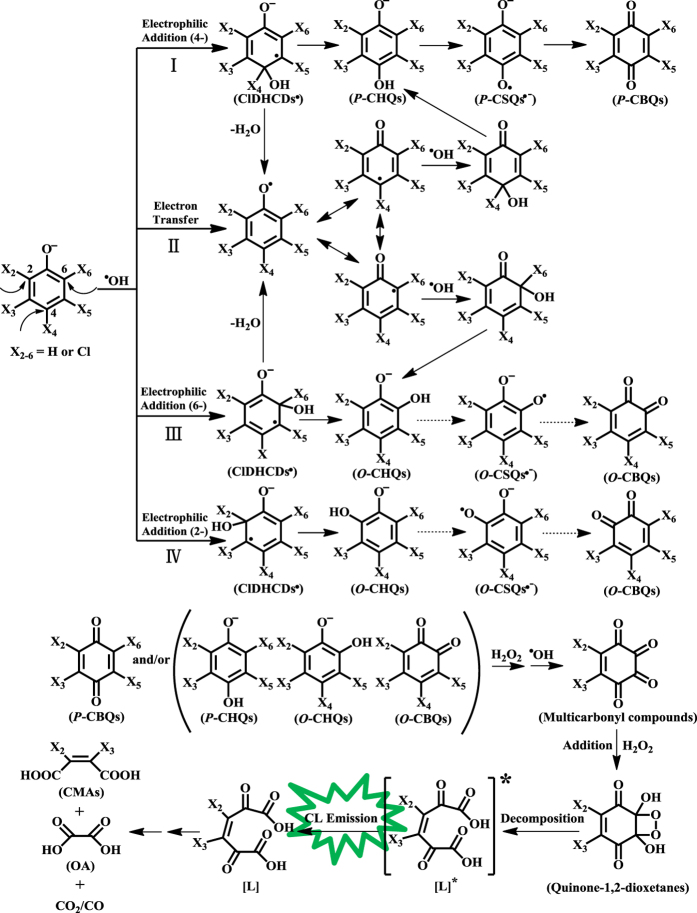
Proposed molecular mechanism for ^•^OH-dependent CL production by the highly chlorinated phenols and Fenton reagent. Dashed lines indicate a possible pathway involving *O*-CSQs^•−^ and *O*-CBQs which were not observed in this study.

On the basis of all the above results and our previous findings^1^,^20^,^21^,^22^,^23^,^45^,^46^,^47^, a molecular mechanism was proposed for ^•^OH-dependent CL production by chlorophenols/H_2_O_2_/Fe^2+^-EDTA (Fig. 7): ^•^OH first attacks chlorophenols, via electrophilic addition (pathways I, III and IV) and/or electron transfer (pathway II), forming the initial chloroquinoid intermediates (*P*-CHQs, *P*-CBQs and *O*-CHQs) and radical intermediates (*P*-CSQs^•−^ and chlorophenoxyl radicals (ClPhO^•^)). If the structure for chlorophenols is symmetric, the two pathways (III and IV) of degradation to *O*-CHQs are exactly identical. For example, 2,3,5,6-TeCP and PCP with the symmetrical structure are degraded to only one *O*-CHQs, which are 3,4,6-*O*-TrCHQ and *O*-TCHQ, respectively. However, for other highly chlorinated phenols (i.e., 2,3,4,5-TeCP and 2,3,4,6-TeCP) with asymmetrical structure, they can be degraded to two different *O*-CHQs (*O*-TCHQ/3,4,5-*O*-TrCHQ and 3,4,6-*O*-TrCHQ/3,4,5-*O*-TrCHQ, respectively) via two different pathways (III and IV). Similar to what we proposed recently^23^, the chloroquinoid intermediates may further react with H_2_O_2_ (and/or ^•^OH), through several steps (for details, see Scheme 1 in ref. 23), to form a multi-carbonyl compound, which is an unstable *o*-quinone and may further react with H_2_O_2_ (and/or ^•^OH) to form a highly-energetic quinone-1,2-dioxetane. An alternative pathway might be that the nucleophilic attack of H_2_O_2_ may preferentially occur on the *o*-quinone intermediates like *O*-CBQs, specifically on the carbonyl carbon which is supposed to be the most electrophilic one. Subsequent intramolecular nucleophilic H_2_O_2_ attack would give rise to a dioxetane intermediate similar to that shown in Fig. 7, but with different substitution pattern. If the quinone ring were substituted by more electron-withdrawing groups (-Cl), the chloroquinoid intermediates will be more readily attacked by the nucleophile H_2_O_2_, due to the decrease in the π-electron density of quinone ring. This should be more favorable for the formation of the highly-energetic quinone-1,2-dioxetanes, which would further decompose to form the electronically excited carbonyl species [L]*. The CL is emitted when the electronically excited state of [L]* returns to its ground state [L], and the subsequent decomposition of [L] leads to the formation of the final ring-opened products such as DCMA, CMA, OA, FA and CO_2_ (and/or CO).

In summary, we found that chlorinated quinoid intermediates and semiquinone radicals were produced during the degradation of the three TeCPs and PCP by Fenton reagent, and the yield and type of which were well correlated with their CL generation. This is the first report showing the critical role of quinoid intermediates and semiquinone radicals in CL generation from polychlorinated phenols and Fenton system. Meanwhile, we also found that the detection limits of these chlorophenols in Fenton system are low, and linear ranges for these chlorophenols are wide (Supplementary Table S5). On the basis of the above results and our previous finding^23^,^27^, this novel CL-dependent method can be used not only to monitor real-time degradation kinetics of chlorophenols, but also to detect and measure trace amounts of chlorophenols in both pure and real samples, especially during advanced oxidation processes. These new findings may have broad chemical and environmental implications for future studies on remediation of other halogenated persistent organic pollutants by advanced oxidation processes.

## Methods

### Chemiluminescence (CL) analysis

The CL produced by chlorophenols and Fenton reagent was measured by an ultraweak CL analyzer (Institute of Biophysics, Chinese Academy of Science, China) with a CR-120 red-sensitive photomultiplier tube (PMT, Hamamatsu, Japan); the CL analyzer was operated in pulse mode. The CL determination was carried out in a 3-mL glass cuvette and started by the injection of Fe^2+^-EDTA after chlorophenols solution was mixed with H_2_O_2_. The conerntrations of chlorophenols, H_2_O_2_ and Fe^2+^-EDTA in the final chelex-pretreated phosphate buffer solution were 0.03 mM, 100 mM and 1 mM, respectively. The CL signal was recorded by a computer equipped with a data-acquisition interface. Data acquisition and treatment were performed with BPCL software. The total intensity of CL was integrated during the whole process for CL measurements. The CL emission spectrum was obtained using a set of interference filters, with the wavelengths from 400 to 640 nm, which were placed between the sample cuvette and the photomultiplier tube. During the determination of CL emission wavelength, appropriate corrections were applied for both spectral response of the photomultiplier tube and transmissivity of filters. However, the CL emission spectrum obtained from the CL analyzer could only provide an approximate range, therefore a spectrofluorometer (Varian) set at CL mode was used to obtain a continuous CL spectrum and get a more accurate maximum wavelength.

### Detection and Identification of Chlorinated *p*-Semiquinone Radicals

The spectra and kinetics of chloro-*p*-semiquinone radicals (*P*-CSQs^•−^) formation by chlorophenols (TeCPs and PCP) and Fenton reagent were recorded and monitored by complementary application of both UV-visible spectrometric methods coupled with stopped-flow technique and direct ESR.

### Electron Spin Resonance (ESR) Studies

ESR spectra were recorded either immediately after the interaction between chlorophenols and Fenton reagent, or at indicated time intervals on a Bruker EMX-plus spectrometer operating at 9.84 GHz and a cavity equipped with a Bruker Aquax liquid sample cell. Typical spectrometer parameters were as follows: scan range, 100 G; field set, 3513 G; time constant, 82 ms; scan time, 82 s; modulation amplitude, 1 G; modulation frequency, 100 kHz; receiver gain, 1.00 × 10^5^ and microwave power, 20 mW. The hyperfine splitting constants were measured by using the simulation software WinSim (version 0.96) (NIEHS)^48^.

### UV-Visible Spectral Analysis

UV-visible spectra of the interaction between chlorophenols and Fenton reagent were monitored by a UV-visible spectrophotometer (Beckman DU-800) in Chelex-treated phosphate buffer (0.1 M, pH 7.4) at room temperature. *P*-CSQs^•−^ were obtained from the reaction between corresponding *P*-CBQs and ascorbic acid (Vc) at a 1:1 molar ratio, and the spectra were recorded from 400 nm to 800 nm. The kinetics of *P*-CSQs^•−^ formation by TeCPs and PCP/Fenton system were followed at the corresponding characteristic wavelengths.

### Stopped-Flow Kinetic Analysis

Stopped-flow spectra were collected on a Biologic SFM-300 system equipped with three syringes and capable of sequential mixing, with a high-speed diode array detector. The syringe 1 was filled with the solution of TeCPs or PCP in phosphate buffer (0.1 M, pH 7.4), while the syringe 2 was filled with an aqueous solution containing H_2_O_2_. The solutions of above two syringes were pre-mixed before rapidly mixing with syringe 3 of Fe^2+^-EDTA solution, and the syringe volume ratio for the three reactants was 1:1:1. The resulting reactant mixture was introduced into the optical cell where the temporal change in absorbance was measured. The stopped-flow spectra were recorded from 400 nm to 600 nm, and the kinetics of *P*-CSQs^•−^ formation by TeCPs and PCP/Fenton system were monitored at the corresponding characteristic wavelength.

## Additional Information

**How to cite this article**: Gao, H.-Y. *et al*. Why Does 2,3,5,6-Tetrachlorophenol Generate the Strongest Intrinsic Chemiluminescence among All Nineteen Chlorophenolic Persistent Organic Pollutants during Environmentally-friendly Advanced Oxidation Process? *Sci. Rep.* **6**, 33159; doi: 10.1038/srep33159 (2016).

## Supplementary Material

Supplementary Information
