# Supplementary material for: Why Does 2,3,5,6-Tetrachlorophenol Generate the Strongest Intrinsic Chemiluminescence among All Nineteen Chlorophenolic Persistent Organic Pollutants during Environmentally-friendly Advanced Oxidation Process?
Source: Sci Rep. 2016 Oct 17;6:33159. doi: 10.1038/srep33159 (PMC5066286; doi:10.1038/srep33159)
Supplement: Supplementary Information [file srep33159-s1.pdf]

Supplementary Information

Why Does 2,3,5,6-Tetrachlorophenol Generate the Strongest Intrinsic Chemiluminescence among All Nineteen Chlorophenolic Persistent Organic Pollutants during Environmentally-friendly Advanced Oxidation Process?

Hui-Ying Gao [1,2], Li Mao [1,2], Bo Shao[1,2], Chun-Hua Huang[1,2] & Ben-Zhan Zhu [1,2,3,*]

[1]State Key Laboratory of Environmental Chemistry and Ecotoxicology, Research Center for Eco-Environmental Sciences, Chinese Academy of Sciences, Beijing, P.R. China 100085

[2]University of Chinese Academy of Sciences, Beijing 100049, China

[3]Linus Pauling Institute, Oregon State University, Corvallis, OR 97331, USA. Correspondence and requests for materials should be addressed to B.-Z. Z. (email: bzhu@rcees.ac.cn)

[*]Corresponding author, E-mail: bzhu@rcees.ac.cn

Materials and Methods

Chemicals: Pentachlorophenol (PCP), 2,3,5,6-tetrachlorophenol (2,3,5,6-TeCP) and 17 other chlorinated phenol congeners, tetrachloro-*p*-hydroquinone (*P*-TCHQ), tetrachloro-*p*-benzoquinone (*P*-TCBQ, also called *p*-chloranil), tetrachloro-*o*-hydroquinone (*O*-TCHQ), 3,4,6-trichloro-*o*-hydroquinone (3,4,6-*O*-TrCHQ), 3,4,5-trichloro-*o*-hydroquinone (3,4,5-*O*-TrCHQ), tetrachloro-*o*-benzoquinone (*O*-TCBQ) and 2,5-dichloro-3,6-dihydroxy-1,4-benzoquinone (DDBQ, also called p-chloranilic acid) were commercially available from Aldrich, Sigma, J&K, CanSyn., Chem. Corp. and Alfa-aesar. Dichloromaleic anhydride, diethyl chloromalonate, oxalic acid, formic acid, tetrabutylammonium hydrogen sulfate (TBAHs), hydrogen peroxide (H_2O_2), ascorbic acid, dimethylsulfoxide (DMSO), benzoate, L-cysteine, trifluoroacetic acid and dimethylformamide (DMF) were purchased from Sigma. HPLC-grade methanol and acetonitrile were obtained from J&K. Trichloro-*p*-benzoquinone (*P*-TrCBQ), and trichlorohydroxy-1,4-benzoquinone (TrCBQ-OH) were synthesized according to the published methods.[1,2] Dichloromaleic acid (DCMA) and chloromalonic acid (CMA) were synthesized by hydrolysis of the corresponding dichloromaleic anhydride and diethyl chloromalonate in water, respectively. All phosphate buffer solutions were Chelex-treated overnight. The chemicals were used as received without further purification.

All chloro-*p*-benzoquinones (*P*-CBQs), *P*-TCHQ and chloro-*o*-hydroquinones (*O*-CHQs), were used to facilitate the identification of the reaction intermediates. Trichloro-*p*-hydroquinone (*P*-TrCHQ) is quantified by HPLC analysis through reduction of the corresponding *P*-TrCBQ. Good efficiency of the conversion of *P*-CBQs to the corresponding chloro-*p*-hydroquinone (*P*-CHQs) by ascorbic acid was confirmed by comparing the HPLC signal obtained from commercially available *P*-TCHQ with the signal of reduced *P*-TCBQ.

HPLC analysis: The reactants (TeCPs and PCP), main reaction intermediates (chloro-*p*-benzoquinones (*P*-CBQs), chloro-*p*-hydroquinones (*P*-CHQs), chloro-*o*-hydroquinones (*O*-CHQs), TrCBQ-OH and DDBQ) and ring-opened product (DCMA) were identified by HPLC equipped with a photodiode array detector (1200, Agilent) including an Eclipse Plus C18 column (15 cm×4.6 mm, 3.5 μm; Agilent). The decay of chlorophenols and the formation of main

intermediates including *P*-CBQs, *P*-CHQs and *O*-CHQs, were analyzed with a mobile phase of methanol and 1% acetic acid at a flow rate of 1.0 mL/min.

TrCBQ-OH and DDBQ formed during the reaction between TeCPs/PCP and Fenton reagent were quantified by HPLC. The mobile phase consists of solvent A (10 mM sodium phosphate buffer containing 5 mM of the ion-paring reagent TBAHs, pH 6.8) and solvent B (methanol). Optimal separation was achieved at A:B = 50:50 (vol/vol) for TrCBQ-OH (295 nm) and A:B = 68:32 (vol/vol) for DDBQ (332 nm) at a flow rate of 1.0 mL/min.

The formation of ring-opened product DCMA was quantified by HPLC with a mobile phase consisting of water (with 0.05% TFA)-methanol (65:35, vol/vol) and UV detection at 245 nm at a flow rate of 0.5 mL/min.

Fluorescence measurement: Fluorescence detection was performed on a Thermo Scientific Varioskan Flash (Thermo Fisher Scientific, USA) spectrofluorometer. The kinetics of $^{\bullet}OH$ formation in 2,3,5,6-TeCP/Fenton system were monitored by fluorescence. Samples with terephthalic acid (TPA) as the $^{\bullet}OH$ probe[3] were excited at 320 nm, and the resulting fluorescence was measured at 425 nm. The basic system consisted of 0-2 mM 2,3,5,6-TeCP, 0-1 mM H_2O_2, 0-5 mM Fe^{2+}-EDTA and 10 mM TPA in Chelex-treated phosphate buffer (0.1 M, pH 7.4) at room temperature.

Ion chromatography: The small molecule ring-opened products and chlorine ion release of chlorophenols after their interaction with Fenton reagent were identified and quantified by a Dionex ICS-1500 ion chromatograph using a Dionex IonPac AS9-HC column with a 9 mM Na_2CO_3 as eluents at a flow rate of 1.0 mL/min. The reaction mixture (TeCPs and PCP, 1 mM; H_2O_2, 300 mM; Fe^{2+}-EDTA, 3 mM) was quenched with catalase and diluted 10 times before IC analysis.

Total organic carbon (TOC) analysis: The inorganic product CO_2 (and/or CO) generated in the reaction between chlorophenols and Fenton reagent was analyzed by the TOC method. The reaction solution was mixed together with Fenton reagent for 5, 10, 20, 30 and 60 min in Chelex-treated phosphate buffer (0.1 M, pH 7.4) at room temperature. TOC determination was carried out by Phoenix 8000 UV-Persulfate TOC analyzer (Tekmar-Dohrmann Co. USA).

References

1. Zhu, B. Z., Kalyanaraman, B. & Jiang, G. B. Molecular mechanism for metal-independent production of hydroxyl radicals by hydrogen peroxide and halogenated quinones. *Proc. Natl. Acad. Sci. U.S.A.* **104**, 17575-17578 (2007).
2. Saby, C., Male, K. B. & Luong, J. H. T. A combined chemical and electrochemical approach using bis(trifluoroacetoxy)iodobenzene and glucose oxidase for the detection of chlorinated phenols. *Anal. Chem.* **69**, 4324-4330 (1997).
3. Barreto, J. C. *et al.* Terephthalic acid: A dosimeter for the detection of hydroxyl radicals in vitro. *Life Sci.* **56**, PL89-PL96 (1994).

Supplementary Figures

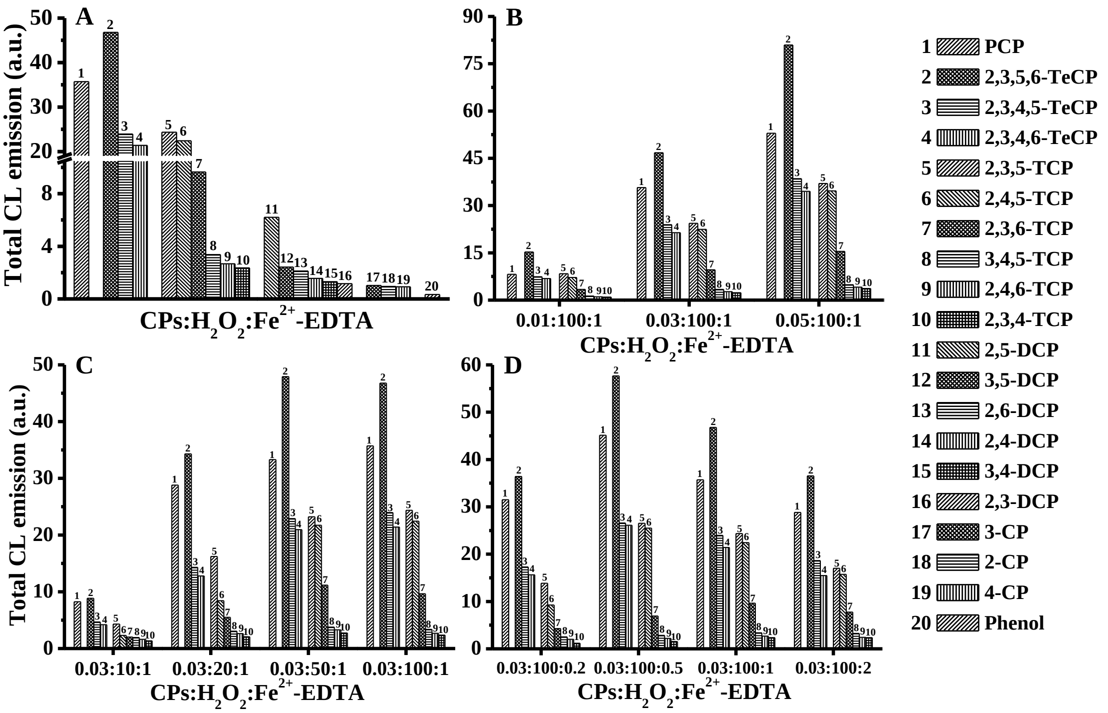

Figure S1. The trend of CL emission from chlorophenols/Fenton system under various different experimental conditions. (A) The trend of CL of 19 chlorophenols in Fenton system. Chlorophenols, 0.03 mM; H_2O_2, 100 mM; Fe^{2+}-EDTA, 1 mM. (B-D) The dose-dependent effect of chlorophenols, H_2O_2 and Fe^{2+}-EDTA on the trend of CL emission of the highly chlorinated phenols including tri-, tetra- and penta-chlorophenols in Fenton system. Chlorophenols, 0.01-0.05 mM; H_2O_2, 10-100 mM; Fe^{2+}-EDTA, 0.2-2 mM. All reactions were carried out in chelex-pretreated phosphate buffer (0.1 M, pH 7.4) at 25 ℃.

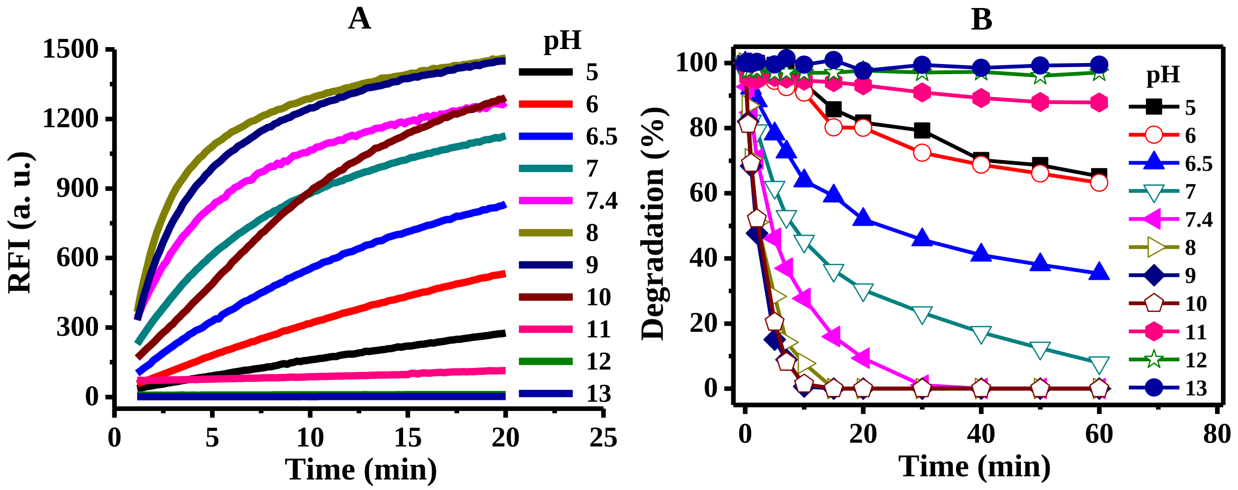

Figure S2. A good correlation between •OH production and 2,3,5,6-TeCP degradation in 2,3,5,6-TeCP/Fenton system. (A) The effect of pH on •OH production by 2,3,5,6-TeCP/Fenton system. pH of phosphate buffer, 5-13; 2,3,5,6-TeCP, 0.03 mM; H_2O_2, 100 mM; Fe^{2+}-EDTA, 1 mM; TPA, 10 mM. (B) The effect of pH on 2,3,5,6-TeCP degradation in 2,3,5,6-TeCP/Fenton system. pH of phosphate buffer, 5-13; 2,3,5,6-TeCP, 0.3 mM; H_2O_2, 100 mM; Fe^{2+}-EDTA, 1 mM. All reactions were carried out in chelex-pretreated phosphate buffer (0.1 M, pH 5-13) at 25 ℃.

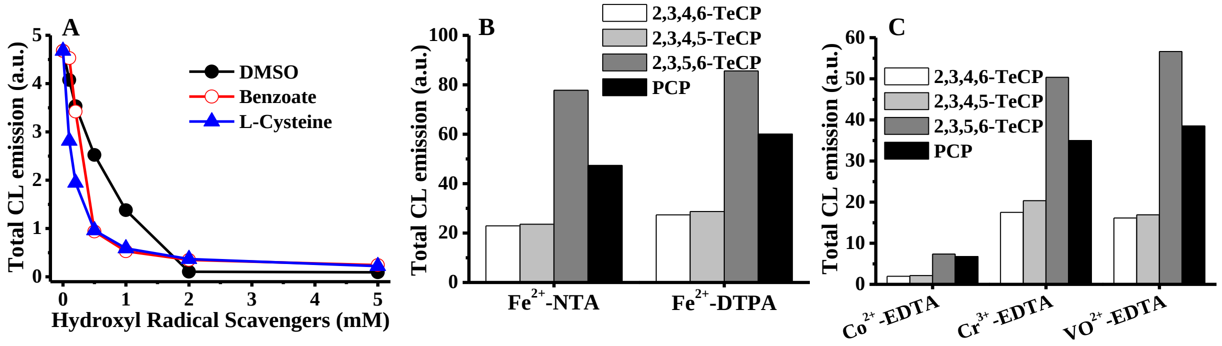

Figure S3. CL produced by TeCPs and PCP/Fenton system was directly dependent on •OH formation. (A) The effect of several classic •OH scavengers on CL produced by 2,3,5,6-TeCP/Fenton system. 2,3,5,6-TeCP, 0.03 mM; H_2O_2, 100 mM; Fe^{2+}-EDTA, 1 mM; DMSO, 0.1-5 mM; Benzoate, 0.1-5 mM; L-Cysteine, 0.1-5 mM. (B and C) CL could also be produced when TeCP and PCP was treated with other •OH-generating systems. (B) Two other Fenton agents Fe^{2+}-DTPA and Fe^{2+}-NTA: TeCPs and PCP, 0.03 mM; H_2O_2, 100 mM; Fe^{2+} complexes, 1 mM.

(C) Fenton-like systems: TeCPs and PCP, 0.03 mM; H_2O_2, 100 mM; VO^{2+}-EDTA, Cr^{3+}-EDTA, Co^{2+}-EDTA, 1 mM. All reactions were carried out in chelex-pretreated phosphate buffer (0.1 M, pH 7.4) at 25 ℃. VO^{2+}, vanadyl(II); Cr^{3+}, chromium(III); Co^{2+}, cobalt(II).

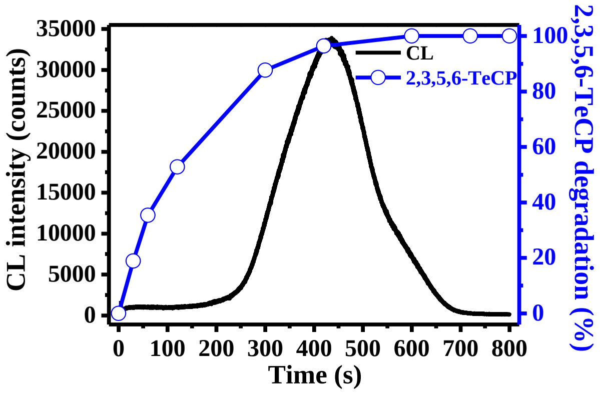

Figure S4. A Good correlation was observed between CL emission and degradation kinetics during advanced oxidation of 2,3,5,6-TeCP. 2,3,5,6-TeCP, 1 mM; H_2O_2, 300 mM; Fe^{2+}-EDTA, 3 mM. The reactions were carried out in chelex-pretreated phosphate buffer (0.1 M, pH 7.4) at 25 ℃.

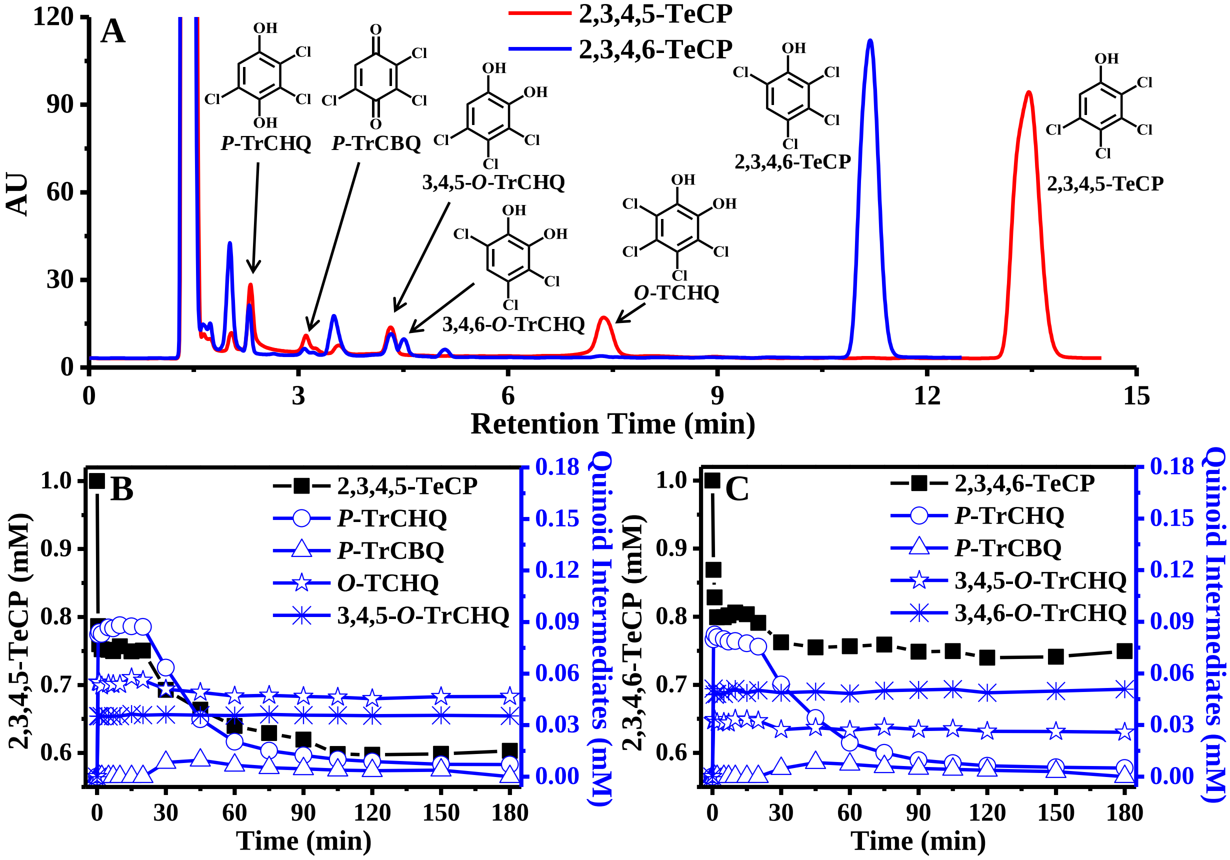

Figure S5. Identification (A) and quantification (B, C) of the major chlorinated quinoid intermediateds from the reaction of 2,3,4,5-TeCP and 2,3,4,6-TeCP with the Fenton system by HPLC analysis. For the separation and identification of chlorophenols and chlorinated quinoid intermediates: 2,3,4,5-/2,3,4,6-TeCP, 1 mM; H_2O_2, 1 mM; Fe^{2+}-EDTA, 3 mM. All reactions were carried out in chelex-pretreated phosphate buffer (0.1 M, pH 7.4) at 25 ℃. 2,3,4,5-TeCP, 2,3,4,5-tetrachlorophenol; 2,3,4,6-TeCP, 2,3,4,6-tetrachlorophenol; *P*-TrCHQ, trichloro-*p*-hydroquinone; *P*-TrCBQ, trichloro-*p*-benzoquinone; *O*-TCHQ, tetrachloro-*o*-hydroquinone; 3,4,6-*O*-TrCHQ, 3,4,6-trichloro-*o*-hydroquinone; 3,4,5-*O*-TrCHQ, 3,4,5-trichloro-*o*-hydroquinone.

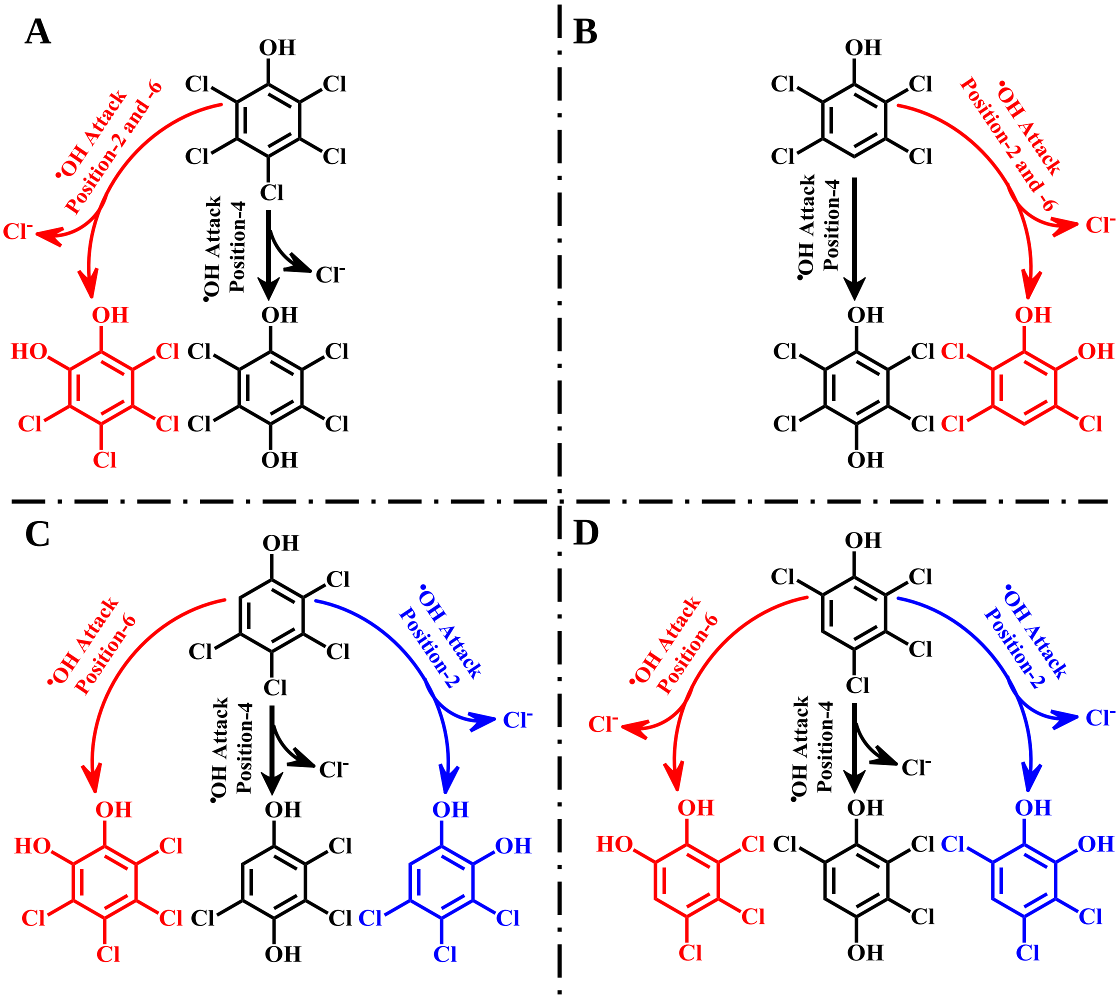

Figure S6. The formation of three kinds of chlorinated quinoid intermediates as determined by the directing effect of -OH group during the degradation of TeCPs and PCP by Fenton system. The different color represents the different pathway of quinone intermediates formation.

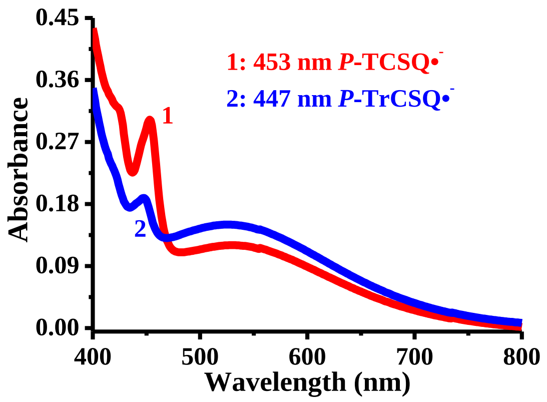

Figure S7. UV-visible absorption spectra of *P*-CSQs$^{\bullet -}$ produced by TeCPs and PCP/Fenton system. TeCPs and PCP, 0.5 mM; H_2O_2, 0.5 mM; Fe^{2+}-EDTA, 1.5 mM. The reactions were carried out in chelex-pretreated phosphate buffer (0.1 M, pH 7.4) at 25 ℃.

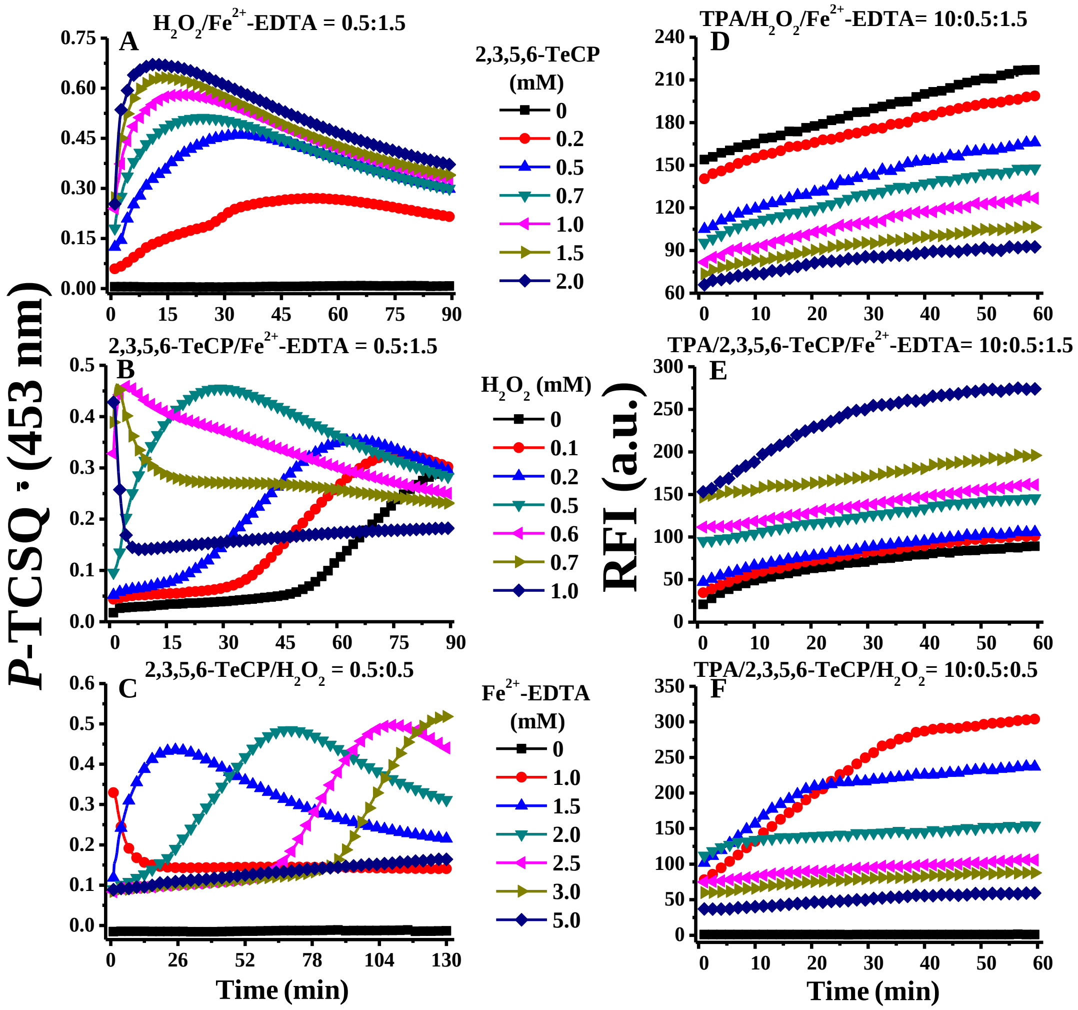

Figure S8. A Good correlation between the formation of *P*-TCSQ$^{\bullet -}$ and $^{\bullet}$OH generation during the degradation of 2,3,5,6-TeCP with Fenton reagent. (A-C) The dose-dependent effects of 2,3,5,6-TeCP, H_2O_2 and Fe^{2+}-EDTA on *P*-TCSQ$^{\bullet -}$ formation by 2,3,5,6-TeCP/Fenton system. *P*-TCSQ$^{\bullet -}$ formation was monitored at 453 nm. 2,3,5,6-TeCP, 0-2 mM; H_2O_2, 0-1 mM; Fe^{2+}-EDTA, 0-5 mM. (D-F) The dose-dependent effects of 2,3,5,6-TeCP, H_2O_2 and Fe^{2+}-EDTA on $^{\bullet}$OH production by 2,3,5,6-TeCP/Fenton by fluorescent method. 2,3,5,6-TeCP, 0-2 mM; H_2O_2, 0-1 mM; Fe^{2+}-EDTA, 0-5 mM; TPA, 10 mM. All reactions were carried out in chelex-pretreated phosphate buffer (0.1 M, pH 7.4) at 25 ℃.

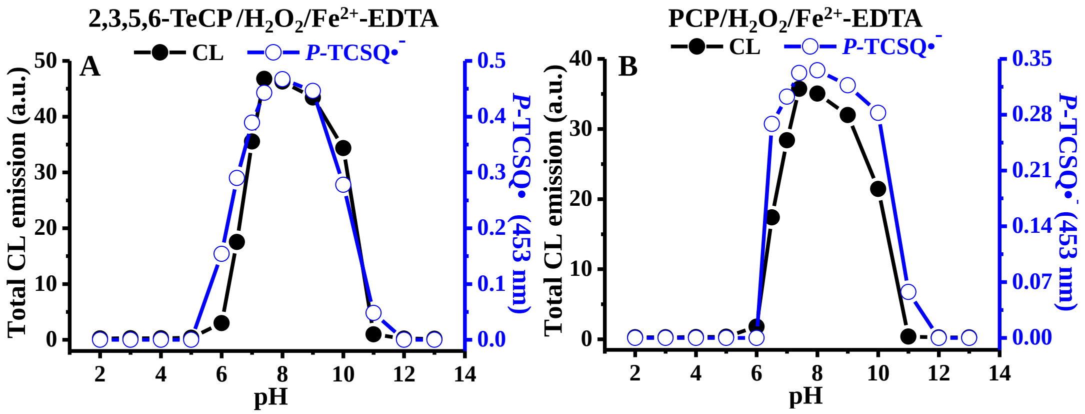

Figure S9. The pH-dependent CL generation and *P*-TCSQ$^{\bullet-}$ production by 2,3,5,6-TeCP and PCP/Fenton system. The reaction mixtures of CL production contained 0.03 mM 2,3,5,6-TeCP and PCP, 100 mM H_2O_2 and 1 mM Fe^{2+}-EDTA. The reaction mixtures of *P*-TCSQ$^{\bullet-}$ formation contained 0.5 mM 2,3,5,6-TeCP and PCP, 0.5 mM H_2O_2 and 1.5 mM Fe^{2+}-EDTA. All reactions were carried out in chelex-pretreated phosphate buffer (0.1 M, pH 7.4) at 25 ℃.

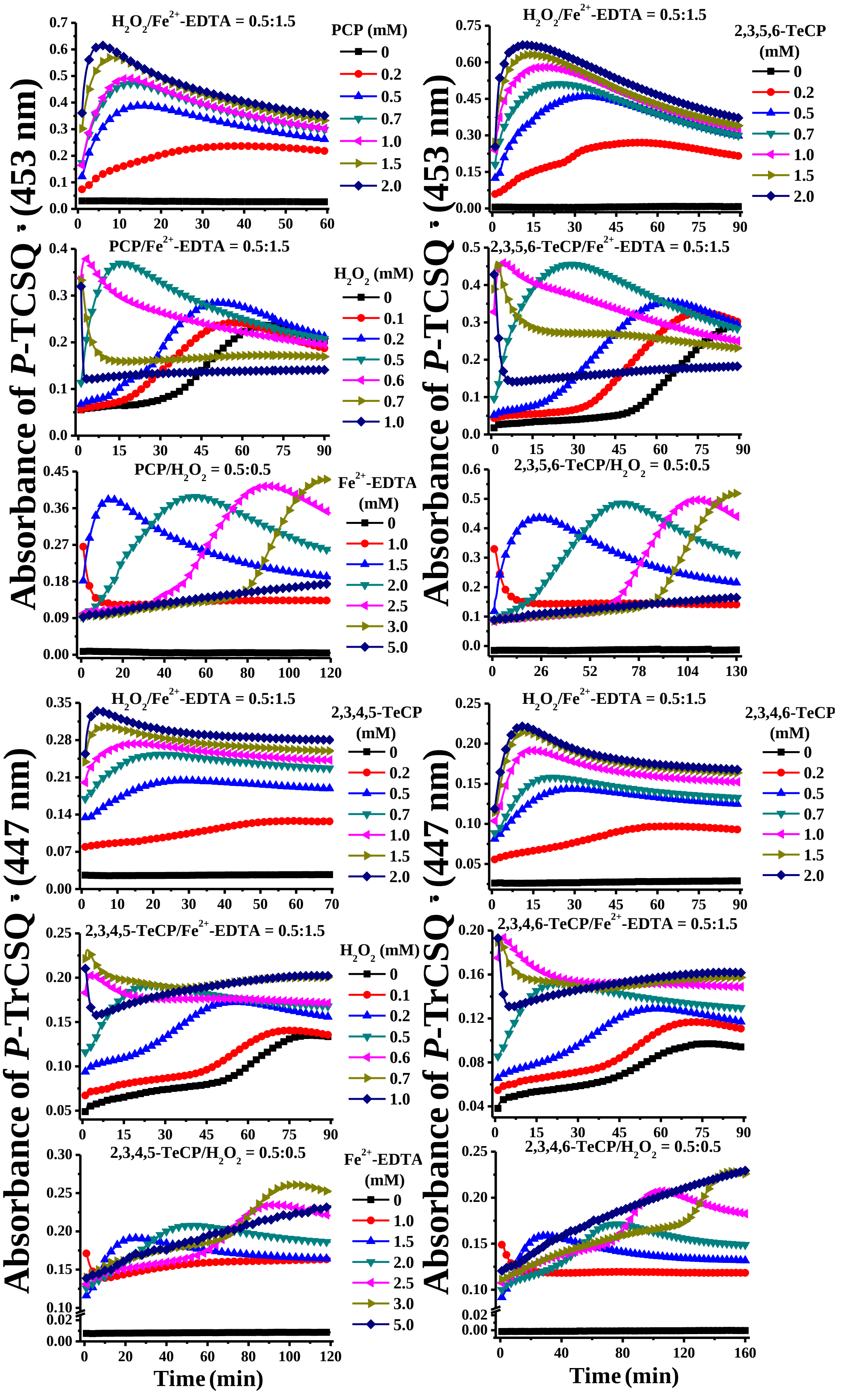
H2O2/Fe2+-EDTA = 0.5:1.5
PCP (mM)
0
0.2
0.5
0.7
1.0
1.5
2.0
2,3,5,6-TeCP (mM)
PCP/Fe2+-EDTA = 0.5:1.5
H2O2 (mM)
0
0.1
0.2
0.5
0.6
0.7
1.0
2,3,5,6-TeCP/Fe2+-EDTA = 0.5:1.5
PCP/H2O2 = 0.5:0.5
Fe2+-EDTA (mM)
0
1.0
1.5
2.0
2.5
3.0
5.0
2,3,5,6-TeCP/H2O2 = 0.5:0.5
Absorbance of P-TCSQ• (453 nm)
2,3,4,5-TeCP (mM)
2,3,4,6-TeCP (mM)
2,3,4,5-TeCP/Fe2+-EDTA = 0.5:1.5
2,3,4,6-TeCP/Fe2+-EDTA = 0.5:1.5
2,3,4,5-TeCP/H2O2 = 0.5:0.5
2,3,4,6-TeCP/H2O2 = 0.5:0.5
Absorbance of P-TrCSQ• (447 nm)
Time (min)

Figure S10. The dose-dependent effects of TeCPs and PCP, H_2O_2 and Fe^{2+}-EDTA on the formation of corresponding *P*-CSQs$^{•-}$ by TeCPs and PCP/Fenton. TeCPs and PCP, 0-2 mM; H_2O_2, 0-1 mM; Fe^{2+}-EDTA, 0-5 mM. All reactions were carried out in chelex-pretreated phosphate buffer (0.1 M, pH 7.4) at 25 ℃.

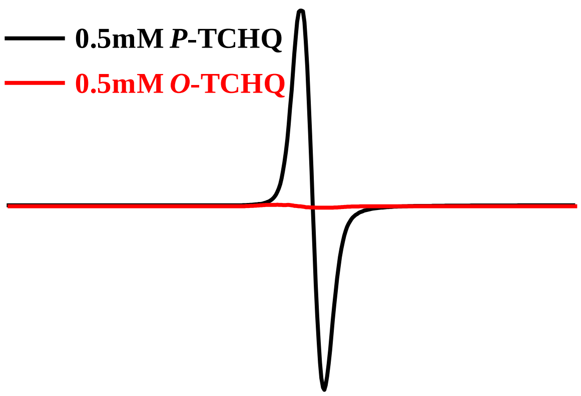

Figure S11. ESR spectra of *P*-TCSQ$^{•-}$ and *O*-TCSQ$^{•-}$ during the oxidation of the same concentration of *P*-TCHQ and *O*-TCHQ by cupric-bathocuproine disulfonate complex (Cu(II)(BCS)$_2$), respectively. *P*-TCHQ/*O*-TCHQ, 0.5 mM; Cu(BCS)$_2$, 0.5 mM. All reactions were carried out in chelex-pretreated phosphate buffer (0.1 M, pH 7.4) at 25 ℃.

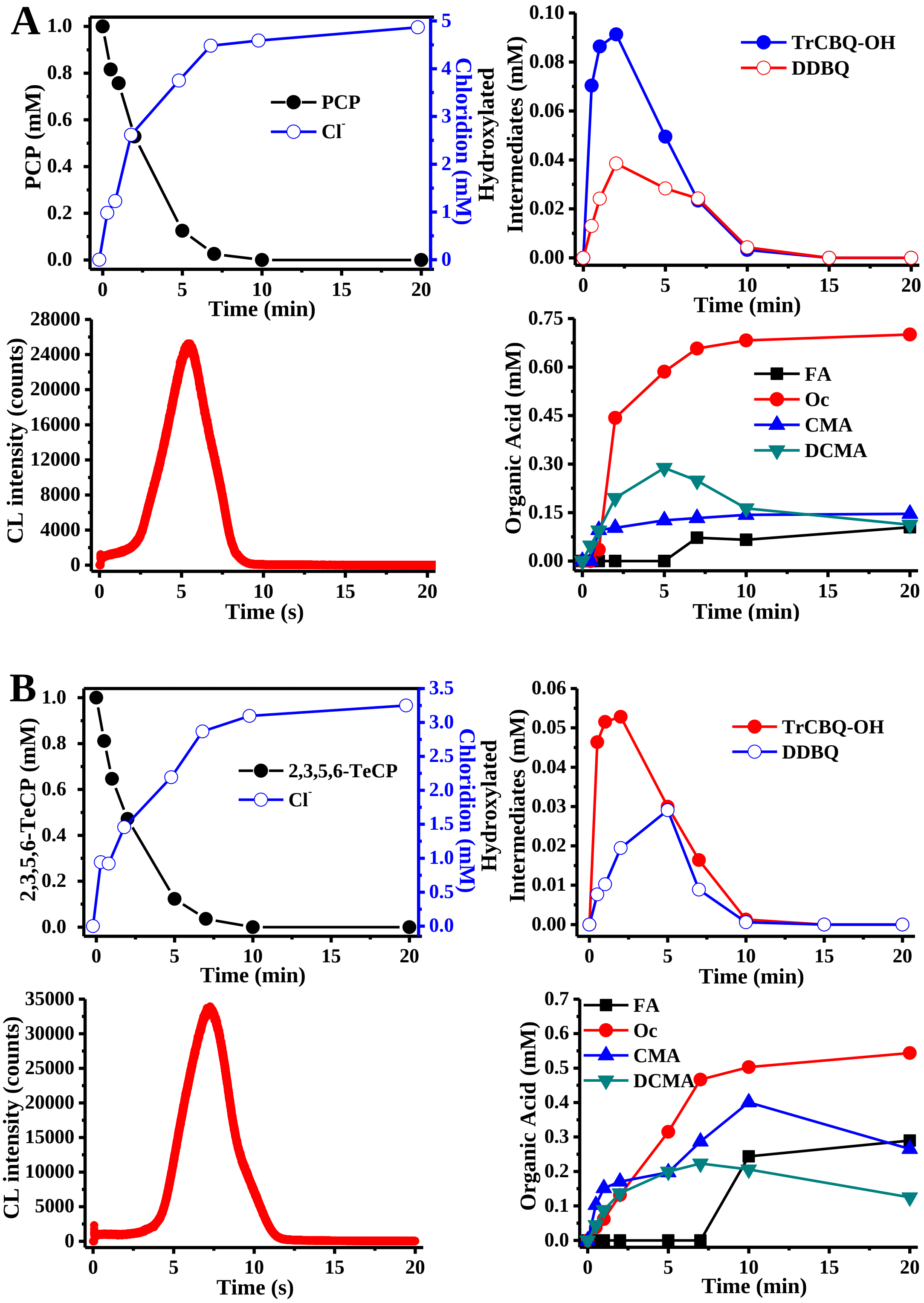
A
B
PCP (mM)
Chloridion (mM)
PCP
Cl-
Time (min)
Hydroxylated Intermediates (mM)
TrCBQ-OH
DDBQ
CL intensity (counts)
Time (s)
Organic Acid (mM)
FA
Oc
CMA
DCMA
2,3,5,6-TeCP (mM)
2,3,5,6-TeCP

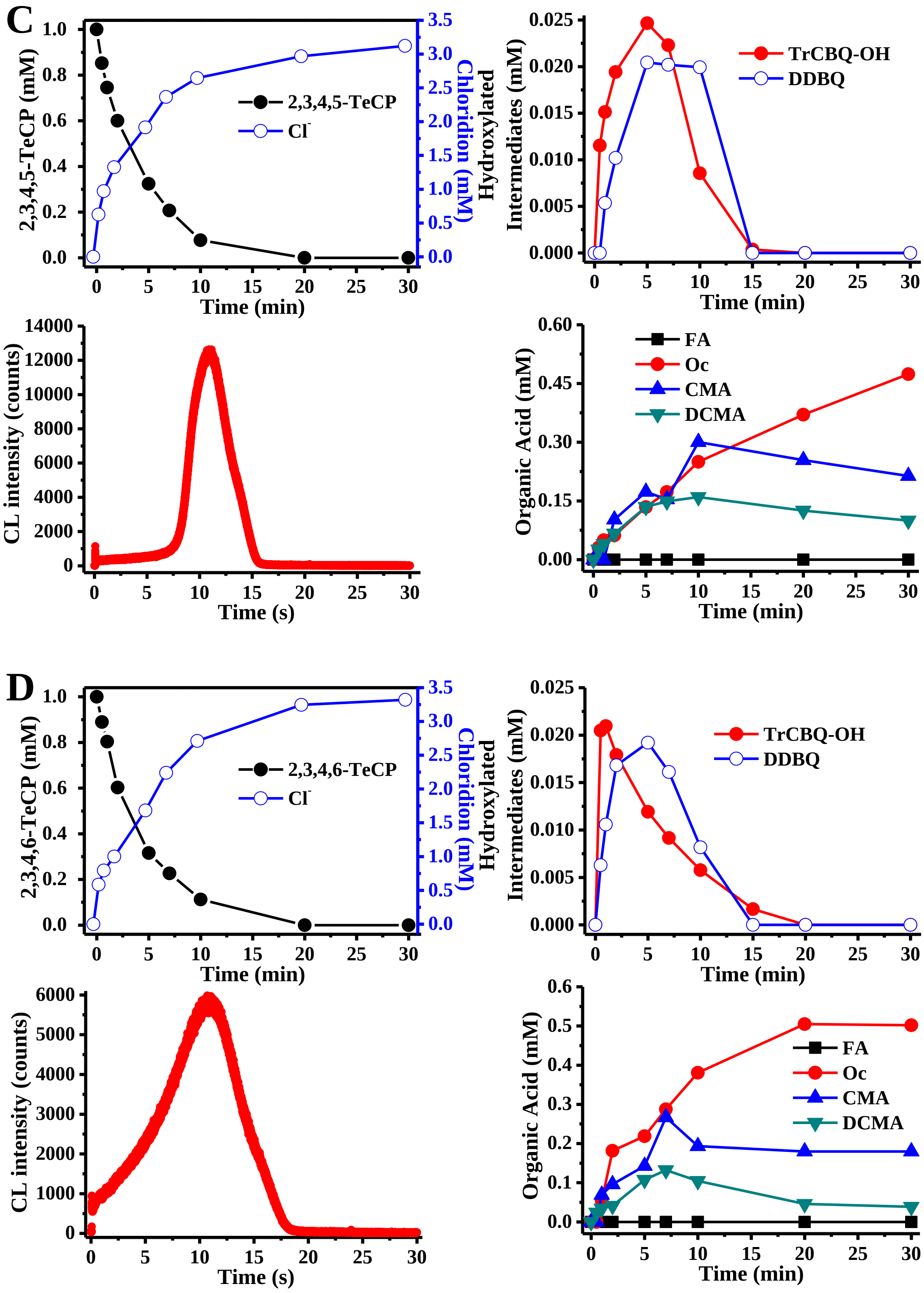

Figure S12. Time course of the formation and decay of TrCBQ-OH, DDBQ and the ring-opened products during the degradation of TeCPs and PCP in Fenton reagent. TeCPs and

PCP, 1 mM; H_2O_2, 300 mM; Fe^{2+}-EDTA, 3 mM. All reactions were carried out in chelex-pretreated phosphate buffer (0.1 M, pH 7.4) at 25 ℃.

Supplementary Tables

Table S1. Maximum concentrations of the major chlorinated quinoid intermediates detected during the degradation of corresponding chlorophenols with Fenton reagent

Chlorophenols	Chloroquinoid Intermediates (mM)							
	P-CBQs		*P*-CHQs		*O*-CHQs			
**PCP**	***P*-TCBQ**	0.024	***P*-TCHQ**	0.109	***O*-TCHQ**	0.068		
**2,3,5,6-TCP**	***P*-TCBQ**	0.095	***P*-TCHQ**	0.151	**3,4,6-*O*-TrCHQ**	0.110		
**2,3,4,5-TCP**	***P*-TrCBQ**	0.010	***P*-TrCHQ**	0.088	***O*-TCHQ**	0.058	**3,4,5-*O*-TrCHQ**	0.036
**2,3,4,6-TCP**	***P*-TrCBQ**	0.008	***P*-TrCHQ**	0.083	**3,4,6-*O*-TrCHQ**	0.051	**3,4,5-*O*-TrCHQ**	0.033

The reaction mixtures contained 1 mM TeCPs and PCP, 1 mM H_2O_2, and 3 mM Fe^{2+}-EDTA.
All reactions were carried out in chelex-pretreated phosphate buffer (0.1 M, pH 7.4) at 25 ℃.

Table S2. Two basic principles responsible for the CL trend of chlorophenols

Chlorophenols	Principle Ⅰ		Principle Ⅱ	CL
	Type of Quinone Intermediates		Total Yield	
	P-TCBQ/*P*-TCHQ	3,4,6-*O*-TrCHQ or *O*-TCHQ		
**PCP**	**Yes**	**Yes**	**0.201**	**35.72**
**2,3,5,6-TeCP**	**Yes**	**Yes**	**0.356**	**46.79**
**2,3,4,5-TeCP**	**NO**	**Yes**	**0.058**	**23.96**
**2,3,4,6-TeCP**	**NO**	**Yes**	**0.051**	**21.41**

Table S3. Charge distribution in chlorophenols from the B3LYP/6-311+G* method

Chlorophenols	Position	Partial charge
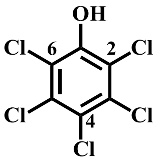	2	0.200
	4	0.112
	6	0.224
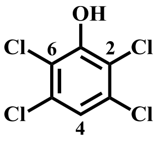	2	0.168
	4	-0.311
	6	0.193
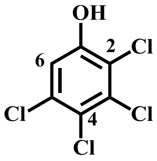	2	0.139
	4	0.078
	6	-0.213
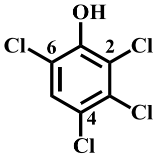	2	0.232
	4	0.246
	6	0.379

Table S4. ESR spectra and *g* values of *P*-CSQs$^{\bullet-}$ produced during the degradation of TeCPs and PCP with Fenton reagent

CPs	ESR Spectra	*P*-CSQs$^{\bullet-}$	a^H	*g* Value
PCP<br>2,3,5,6-TeCP	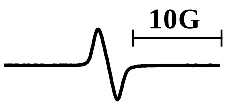	*P*-TCSQ$^{\bullet-}$	-	2.0054
2,3,4,5-TeCP<br>2,3,4,6-TeCP	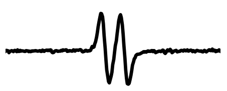	*P*-TrCSQ$^{\bullet-}$	2.17	2.0051

The reaction mixtures contained 0.5 mM TeCPs and PCP, 0.5 mM H_2O_2, and 1.5 mM Fe^{2+}-EDTA.
All reactions were carried out in chelex-pretreated phosphate buffer (0.1 M, pH 7.4) at 25 ℃.

Table S5. Linear range and detection limit for TeCPs and PCP as measured by the CL method developed in this study

Chlorophenols	Detection limit (μM)	Linear range (μM)	R^2
**2,3,4,6-TeCP**	0.01	0.03～100	0.9998
**2,3,4,5-TeCP**	0.01	0.03～100	0.9997
**2,3,5,6-TeCP**	0.007	0.01～100	0.9997
**PCP**	0.007	0.01～100	0.9989

The reaction mixtures contained 100 mM H_2O_2, and 1 mM Fe^{2+}-EDTA.
All reactions were carried out in chelex-pretreated phosphate buffer (0.1 M, pH 7.4) at 37 ℃.
